# Presynaptic dysregulation of the paraventricular thalamic nucleus causes depression-like behavior

**DOI:** 10.1038/s41598-019-52984-y

**Published:** 2019-11-11

**Authors:** Tomoaki M. Kato, Noriko Fujimori-Tonou, Hiroaki Mizukami, Keiya Ozawa, Shigeyoshi Fujisawa, Tadafumi Kato

**Affiliations:** 1 Laboratory for Molecular Dynamics of Mental Disorders, RIKEN Center for Brain Science, Wako, Saitama, Japan; 2 Division of Genetic Therapeutics, Center for Molecular Medicine, Jichi Medical University, Shimotsuke-shi, Tochigi, Japan; 3 Laboratory for Systems Neurophysiology, RIKEN Center for Brain Science, Wako, Saitama, Japan; 4 Present Address: Department of Fundamental Cell Technology, Center for iPS Cell Research and Application, Kyoto University, Kyoto, Japan

**Keywords:** Depression, Depression

## Abstract

The paraventricular thalamic nucleus (PVT) is a part of epithalamus and sends outputs to emotion-related brain areas such as the medial prefrontal cortex, nucleus accumbens, and amygdala. Various functional roles of the PVT in emotion-related behaviors are drawing attention. Here, we investigated the effect of manipulation of PVT neurons on the firing patterns of medial prefrontal cortical (mPFC) neurons and depression-like behavior. Extracellular single-unit recordings revealed that acute activation of PVT neurons by hM3Dq, an activation type of designer receptors exclusively activated by designer drugs (DREADDs), and administration of clozapine N-oxide (CNO) caused firing rate changes in mPFC neurons. Moreover, chronic presynaptic inhibition in PVT neurons by tetanus toxin (TeTX) increased the proportion of interneurons among firing neurons in mPFC and shortened the immobility time in the forced swimming test, whereas long-term activation of PVT neurons by hM3Dq caused recurrent hypoactivity episodes. These findings suggest that PVT neurons regulate the excitation/inhibition balance in the mPFC and mood stability.

## Introduction

The paraventricular thalamic nucleus (PVT) is a part of epithalamus having characteristic neural connections. The PVT receives input from serotonergic neurons, CRH (corticotoropin-releasing hormone) neurons in the hypothalamus, and suprachiasmatic nucleus. On the other hand, it sends output to the medial prefrontal cortex, nucleus accumbens, amygdala, and insular^1^. Thus, the PVT is connected with many of brain regions implicated in emotion regulation. Recent studies show various emotion-related roles of the PVT such as fear conditioning^2^, opiate withdrawal^3^, saliency^4^, and wakefulness^5^.

We recently reported that neuron-specific transgenic mice with mutant polymerase γ (*Polg*), a causative gene for mitochondrial diseases, showed recurrent spontaneous depression-like episodes and accumulation of deleted mitochondrial DNA (mtDNA) in the PVT^6^, suggesting the possible role of the PVT in mood regulation^7^. However, the role of the PVT in mood regulation has not been extensively studied yet. Though the output from the PVT to the nucleus accumbens^3^ and amygdala^2^ has been extensively studied, the role of the efferent projections from the PVT to the medial prefrontal cortex, which plays a central role in depression-like behavior, has not been well studied yet.

In this study, we first investigated the neurophysiological effect of PVT manipulation on mPFC neurons by using genetic and pharmacogenetic approaches. We generated mice infected with adeno-associated virus (AAV) expressing the designer receptors exclusively activated by designer drugs (DREADDs) hM3Dq and Cre recombinase to induce tetanus toxin (TeTX), which cleaved the Vamp2 protein required for synaptic transmission, and then obtained electrophysiological recordings. Thereafter, we evaluated the effects of acute or long-term hM3Dq- or hM4Di-, a Gi-coupled inhibitory DREADD, and TeTX-PVT manipulation on the behavior in the long-term measurements of spontaneous wheel-running activity, forced swimming test (FST) and tail suspension test (TST).

## Materials and Methods

### Animals

All animal care and experimental procedures were in accordance with the guidelines for proper conduct of animal experiments published by the Science Council of Japan, and all the experiments were approved by RIKEN Wako Animal Experiment Committee and RIKEN Genetic Recombinant Experiment Safety Committee. For presynaptic inhibition in specific neurons by TeTX light chain, *CaMKIIα*-promoter-loxP-STOP-loxP-tTA (Tg2) and *TetO*-TeTX (Tg3) transgenic mice, which were kindly provided by Dr. S. Tonegawa (Massachusetts Institute of Technology, Boston, MA, USA), generated under a C57BL/6 genetic background were used^8^. Heterozygous Tg2 and Tg3 were crossed to obtain the double-Tg mutant mice (Tg2/+; Tg3/+) which express TeTX depending on Cre recombinase expression. Other progenies (single mutant of Tg2/+ or Tg3/+, or wild-type+/+) were used as control mice. Behavioral data of the mice were excluded when they were injured or died due to an accident.

### Chemicals

In the experiments for DREADD, clozapine N-oxide (CNO) (3 µg/g body weight, Enzo Life Sciences, Farmingdale, NY, USA) was administered by intraperitoneal injection at least one hour before behavioral experiments and mouse brain fixation. For long-term modulation of neuronal activity by DREADD, slow-releasing pellets of CNO for 90 days (Innovative Research of America, Sarasota, FL, USA) were implanted into the back subcutaneous tissue of the interscapular region under isoflurane anesthesia. Chow containing doxycycline (10 mg/kg, Oriental Yeast Co., Ltd., Tokyo, Japan) was fed to repress the expression of TeTX for at least two weeks.

### Virus and its injection into the PVT

AAV2-hSyn-HA-hM3D(Gq)-IRES-mCitrine, AAV2-hSyn-HA-hM4D(Gi)-IRES-mCitrine, and AAV8-hSyn-DIO-HA-hM3Dq(Gq)-IRES-mCitrine were purchased from UNC Vector Core (University of North Carolina at Chapel Hill, Chapel Hill, NC, USA). A bicistronic expression vector for Cre recombinase and EGFP (AAV2-Cre-IRES-EGFP) was produced as previously described^6,9^. In brief, a PCR fragment containing Cre-IRES-EGFP was subcloned under the pCMV-β globin intron driver of AAV MCS vector to obtain pAAV-Cre-IRES-EGFP. The AAV2-CMV-hrGFP vector was purchased (Agilent, La Jolla, CA, USA). AAV particles were produced by using HEK293 cells. Before the surgery, mice were anesthetized with isoflurane and fixed in a stereotaxic frame. For the electrophysiological experiments, 1.0 µL of virus mixture containing AAV2-Cre-IRES-EGFP and AAV8-hSyn-DIO-HA-hM3Dq(Gq)-IRES-mCitrine (1:4) was injected into the PVT (AP –1.7 mm, ML 0.0 mm, and DV 3.2 mm from the bregma) by using a pump (UMP3; World precision instruments, Sarasota, FL, USA) with a 10-µL Hamilton syringe attached to a 33-gauge needle at the speed of 1 μl/20 min as described in previous paper^6^. For the behavioral tests in the TeTX mice, 1.0 µL of AAV2-Cre-IRES-EGFP was injected in the PVT. Male heterozygous double-Tg mice (28–30 weeks old) Tg2/+;Tg3/+ [N = 8] or their other male littermate controls (N = 5:single mutant of Tg2/+ [N = 2], Tg3/+ [N = 1] and wild-type, +/+ [N = 2]) were used. After wheel-running measurements, the FST and TST were performed at 54–66 week of age, and the mice were sacrificed and the injection sites of AAV were verified by immunohistochemistry at the age of 65–70 weeks. For the behavioral tests in DREADD experiments, male C57BL/6 J mice were purchased from CLEA Japan Ltd. (Tokyo, Japan) at 8 weeks of age and used for the experiments. AAV2-hSyn-HA-hM3D(Gq)-IRES-mCitrine (N = 11), AAV2-hSyn-HA-hM4D(Gi)-IRES-mCitrine (N = 14), and AAV2-hSyn-EGFP (N = 12) were injected at 9–11 weeks of age, as described above. From 26 weeks of age, behavioral tests including the FST and TST were performed. At 48 weeks of age, the mice were sacrificed and the injection sites were verified by immunohistochemistry.

### Electrophysiology

Detailed surgical procedures for chronic recording are described in a previous paper^10^. In brief, a 32-channel silicon probe (A4x2-tet-5mm-500-400-121, NeuroNexus, Ann Arbor, MI, USA) was attached to a micromanipulator to adjust the desired depth position. Three mice were implanted with a silicon probe whose shanks were aligned along the rostrocaudal axis and a 10 degree angle from vertical axis in the mPFC (AP = 1.30 mm, ML = 0.75 mm, DV = 2.0 mm from the bregma) for data acquisition. Ground and reference wires were connected to screws implanted in the bone above cerebellum. During the recording sessions, animals were connected to a multiple channel amplifier (KJE1001, Amplipex Ltd, Hungary) via a headstage amplifier fixed to the micromanipulator to obtain neurophysiological signals at 20 kHz sampling rate and were allowed to freely move in an acrylic box (30 cm width × 30 cm height × 30 cm depth) covered with aluminum foil and white paper for animal bedding. Spike sorting was performed semi-automatically with KlustaKwik2^11^, followed by manual adjustment by using Klusters v2.0.0^12^. The clusters that included obvious noise were removed and those that were stable throughout the experiment were used for analysis. For electrophysiological experiments, AAV mixture (described above) was injected to the PVT of Tg2/+; Tg3/+ male mice as described above. Initially, the mice were fed with the chow containing doxycycline. In a single session, at 30 mins after the recording start, CNO or vehicle (phosphate buffered saline, PBS) was intraperitoneally injected into the mice, and the recording was conducted for 120 mins. After that, doxycycline-free chow was given to the mice for 2 weeks, and the same experiments were performed.

### Immunohistochemistry

Mice were deeply anesthetized by intraperitoneal injection of 2.5% avertin (Tribromoethanol) at a dosage rate of 0.2 mL/10 g body weight and were fixed by transcardial perfusion with saline (0.9%), followed by 4% paraformaldehyde (PFA) in 0.1 M phosphate buffer (PB, pH 7.4) and post-fixation with the same fixative overnight. The fixed brains were immersed in 30% sucrose in 0.1 M PB until sinking and embedded in OCT compound (Sakura Finetek Japan Co., Ltd, Tokyo, Japan). Cryosections of 14-µm thickness were prepared on a cryostat and attached to microscope slides. As primary antibodies, rabbit polyclonal anti-c-fos antibody (1:5000, PC38, Calbiochem, San Diego, CA, USA), rabbit polyclonal anti-pCREB antibody (1:500, #9198, Cell Signaling Technology, Danvers, MA, USA), and chicken polyclonal anti-GFP antibody (1:500, #GFP-1020, Aves labs, Tigard, OR, USA) were used. For pCREB staining, signals were enhanced by using TSA biotin system (PerkinElmer Japan Co., Ltd., Kanagawa, Japan). The localization of the antigen was visualized by using donkey anti-rabbit IgG Dylight 594, donkey anti-chicken IgY Dylight 488, donkey anti-rabbit IgG biotin, and streptavidine Dylight 594 as secondary antibodies. All secondary antibodies were purchased from Jackson ImmunoResearch (West Grove, PA, USA). The images were captured by confocal microscopy (FV1000; Olympus, Tokyo, Japan).

### Behavioral analysis

#### Forced swimming test

Behavioral analyses were performed as described previously^6^. In brief, the mouse was placed in a transparent, acrylic cylinder (11 cm diameter × 21.5 cm height) filled with tap water (22.5 °C ± 0.5 °C) with the depth of 11.5 cm. The cylinder was placed in an opaque box (33 cm × 22 cm × 48 cm), and the behavior of the mouse was recorded by a CCD video camera. In the experiments for TeTX mice, we tried to examine the effect of TeTX-on to TeTX-off switching, and the same animals were subjected to the FST again after doxycycline-chow feeding for more than for 2 weeks. However, it was difficult to compare the difference because of the shortened immobility time due to learned helplessness. In this paper, we describe approximately the initial 6 min in the first session. In the DREADD experiments, a single swimming session for 6 min was performed.

#### Tail suspension test

For the TST, the mouse was suspended with its tail attached to a small aluminum plate by adhesive medical tape, and the plate was hooked to an attachment located inside an opaque box (48 × 22 × 33 cm). The behavior of the mouse was recorded by a CCD video camera for 6 min.

### Long-term analysis of wheel running

Wheel-running activity of the female mice was recorded and analyzed as described previously^6^. Each mouse was individually housed in a cage equipped with a running wheel (5 cm wide and 14 cm in diameter) and an automatic counter for wheel rotation (O’hara & Co., Ltd., Tokyo, Japan). We prepared three groups of mice, AAV-hM3Dq (N = 12), AAV-hM4Di (N = 12) and AAV-GFP control (N = 6). Among these, 11 mice (N = 3, N = 4, and N = 4, respectively) did not show typical patterns of wheel running. Some did not learn wheel running, and others showed an abnormal pattern due to equipment issues, resulting in a constant light (LL) condition. These mice were excluded from the behavioral analysis. Because the number of the mice with AAV-GFP was too small to apply statistical analyses, the data of this group were shown only as a reference. At 124 days after the initiation of the wheel running, a pellet containing CNO was implanted. The depression-like episodes were defined by the relative strength index (RSI) as reported previously. Since the test period for the effect of DREADD in this study was shorter (3 months) than our previous study (> = 6 months), we used relaxed criteria for the episodes (minimum 7 days with RSI < 50, instead of 9 days in the original criteria, and at least one day with RSI <30, instead of RSI <25 in the original criteria). When the episode duration overlapped with the day of the start of CNO, episodes with low RSI (<30) seen before the initiation of CNO treatment were included into the period before CNO, and those with the low RSI that appeared only after the initiation of CNO were included into the period after CNO. Even when the original criteria for the definition of the episode were used, the results were basically similar.

### Data analysis and statistics

Statistical analysis was performed using Microsoft Excel (Microsoft Japan, Tokyo, Japan), Prism 4 (GraphPad Software, Inc., La Jolla, CA, USA) and an online website (http://vassarstats.net/). In the experiments for the electrophysiology, firing patterns of well-isolated units were analyzed using a custom script for Matlab with signal processing and statistic toolboxes (Mathworks, Natick, MA)^13^ and Python 3 included in the Anaconda distribution downloaded from https://anaconda.org/. To examine the changes in firing rate (FR) associated with hM3Dq and/or TeTX, the obtained FR from each unit was evaluated as log2 ratio of each quarter to the first quarter in the sessions. Based on the patterns of normalized FR changes, the single units were divided into several clusters by using the “TimeSeriesKmeans” method with the Euclidean metric to calculate distance in the tslearn package in Python 3. The optimal number of clusters was decided by using the “elbow method,” in which distortion was calculated as summation of total distances to the nearest cluster centers. We used eight cluster divisions for further analysis (Supplementary Fig. 1C,D). The significance of FR change was determined if the slopes of the linear regression of the normalized FR in the clusters were not significantly equal to zero (*p < *0.05) and their absolute values were more than 1.0/120 mins in the sessions. Fisher’s exact test was used for analysis of the proportion of the single units. The difference in the average FR at baseline (30 mins before the administration of PBS or CNO) was tested by Student’s t-test. The difference in the proportion of inter-spike intervals (ISIs) was tested by the Kolmogrov-Smirnov (KS) test. When equal variance could not be assumed by Levene’s test, Student t-test without assumption of equal variances was used. In the behavioral analysis, the immobility time and wheel-running activity were processed by the TimeFZ2 software attached to the equipment (O’hara & Co., Ltd.). In the immunohistochemical analysis, the number of c-fos- or pCREB-positive cells was counted by using ImageJ included in the Fiji distribution downloaded from https://fiji.sc/. One-way ANOVA with post-hoc Tukey’s test was used for analysis of the number of c-fos- or pCREB-positive cells and immobility time in DREADD experiments. The one-tailed Student’s t-test was used for the comparison of control and TeTX mice. The Kruskal-Wallis test was used for the comparison of the values obtained before and after CNO pellet implantation in wheel-running experiments.

Statistical analysis was performed as described in Supplementary Methods. All data used for statistical analysis is available upon request.

## Results

### Firing patterns of mPFC neurons after activity modulation of PVT neurons

**Figure 1:**
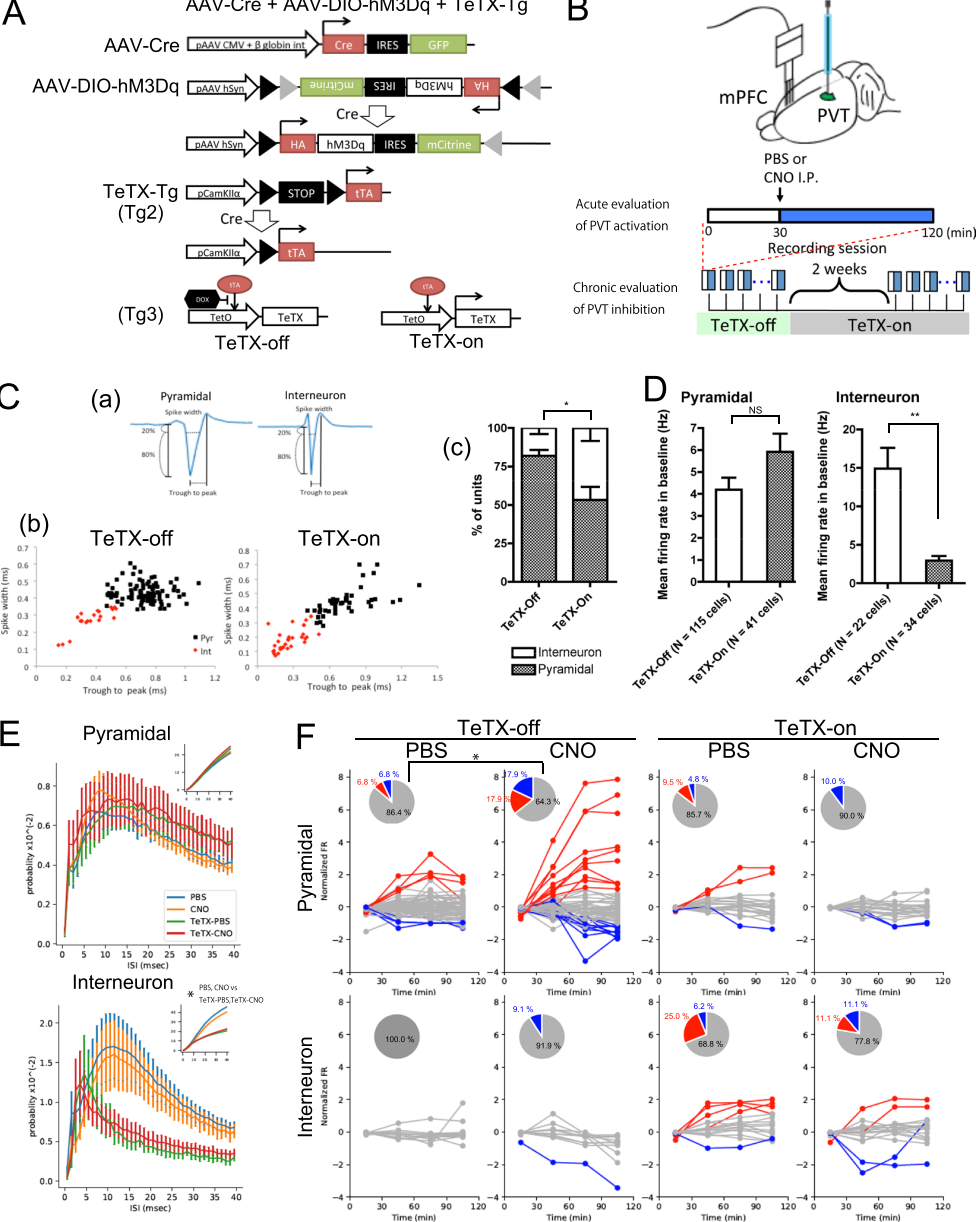
Effect of manipulation of PVT neurons by hM3Dq and TeTX on neuronal activity in the mPFC. (**A**) AAV constructs and genetic background of TeTX mice. After excision of floxed STOP by Cre recombinase, TeTX expression was induced by tTA that was inhibited by doxycycline. TeTX was expressed in doxycycline-free conditions. AAV-hM3Dq with double-floxed inverse open reading frame (DIO) expresses hM3Dq when co-injected with AAV-Cre. (**B**) Schematic drawing of electrophysiological experiments. AAVs were injected into the PVT and electrodes were implanted into the mPFC. Each recording session consisted of first 30 min of baseline and the subsequent 90 min of the test period for acute evaluation of activation of PVT neurons. Recordings were conducted under TeTX-off and TeTX-on conditions to evaluate chronic effect of presynaptic inhibition of PVT neurons. (**C**) (a) Classification of isolated units based on spike waveforms. (b) The two clusters demarcated by a border in the trough-to-peak vs spike width plot indicated rapid-firing putative interneurons (red) and regular-firing pyramidal neurons (black) in the TeTX-off conditions (left) and the TeTX-on conditions (right). (c) The cumulative bar graph indicates the proportion of pyramidal neurons and interneurons in isolated units. Error bars indicate standard error of mean (SEM). * indicates p < 0.05 by two-tailed Student’s t-test. (**D**) Average FR of pyramidal neurons (left) and interneurons (right) during baseline. **indicates *p* < 0.001 and NS indicates *p* > 0.05 by two-tailed Student’s t-test. Error bars indicate SEM. (**E**) Probability distributions of inter-spike intervals (ISIs) of pyramidal neurons (upper) and interneurons (bottom). The probabilities in each 1 ms-bin (main graph) and the cumulative probabilities (inset) in PBS (cyan) or CNO (orange) administration in the TeTX-off conditions or PBS (green) or CNO (red) administration in the TeTX-on conditions are shown. Error bars indicate SEM. * indicates *p* < 0.05 by 2 sample Kolmogorov-Smirnov test. (**F**) Line plots indicate the time course of normalized firing rate (FR) of pyramidal neurons (upper) and interneurons (bottom) in the indicated experimental conditions. Pie charts indicate the proportion of cells classified into clusters showing stable FR (grey), decreasing FR (blue), and increasing FR (red). Colors in line plots also indicate the cells in each cluster. *indicates *p* < 0.05 by two-tailed Fisher’s exact test. Details of clustering of all units are shown Supplementary Fig. 1.

We generated the TeTX transgenic mice that were infected by AAV-Cre and AAV-DIO-hM3Dq and investigated the effect of manipulation of PVT neurons on neuronal activities in the mPFC. These mice enable us to bi-directionally modulate PVT activity by CNO administration for acute activation and by withdrawing doxycycline for chronic inhibition of their synaptic transmission. By using these mice, we conducted extracellular single-unit recordings in the mPFC to examine the effect of acute and chronic activity modulation of PVT neurons by hM3Dq and CNO administration under the TeTX-off or TeTX-on conditions (Fig. 1A,B). In the well-isolated units, there were two typical types of spike forms demarcated by a border in the trough-to-peak and spike width features indicating the rapid-firing putative interneurons (0.386 ± 0.026 ms [mean ± standard error of mean (SEM)]: trough-to-peak, 0.279 ± 0.014 ms: spike width) and regular firing putative pyramidal neurons (0.712 ± 0.012 ms: trough-to-peak, 0.437 ± 0.005 ms: spike width Fig. 1C(a,b))^14^. To evaluate the effect of chronic presynaptic inhibition of PVT neurons, we compared the activity of mPFC neurons during baseline, the first 30 mins in each session, and found that the number of well-isolated units was decreased in the TeTX-on condition, the proportion of interneurons in the units, however, was significantly higher (Fig. 1C(c)). The average FR of interneurons during baseline was significantly reduced in the TeTX-on conditions, whereas that of pyramidal neurons did not change (Fig. 1D). In addition to the reduction of FR, the proportion of inter-spike intervals (ISIs) of interneurons in the TeTX-on conditions was biased toward being shorter than that in the TeTX-off conditions, irrespective of PBS or CNO administration, indicating intermittent firing patterns (Fig. 1E, Supplementary Fig. 1A). These ISIs in the TeTX-on conditions had a tendency to shorten as the sessions progressed (Supplementary Fig. 1B). Next, to evaluate the effect of acute activation of hM3Dq-expressing PVT neurons by CNO administration, we observed time course of FR of pyramidal neurons and interneurons (Fig. 1F). Based on their firing patterns, units were divided into eight clusters and the units in the clusters that showed significant trend were defined as the cell groups showing increasing or decreasing FR (Supplementary Fig. 1C,D). The activities of mPFC pyramidal neurons in the TeTX-off conditions were altered by CNO administration whereas that in interneurons in the TeTX-off conditions and in both cells in the TeTX-on conditions were not, suggesting that neuronal activity changes in the mPFC by PVT activation had been mediated by synaptic transmission from PVT neurons to mPFC neurons. These experiments indicated that synaptic transmission of PVT neurons affected the activity of mPFC pyramidal neurons and chronic presynaptic inhibition in PVT neurons altered the proportion of excitatory and inhibitory neurons in the mPFC and induced intermittent firing patterns with decreased FR in mPFC interneurons.

### Long-term effect of continuous manipulation of PVT neurons by DREADD

**Figure 2:**
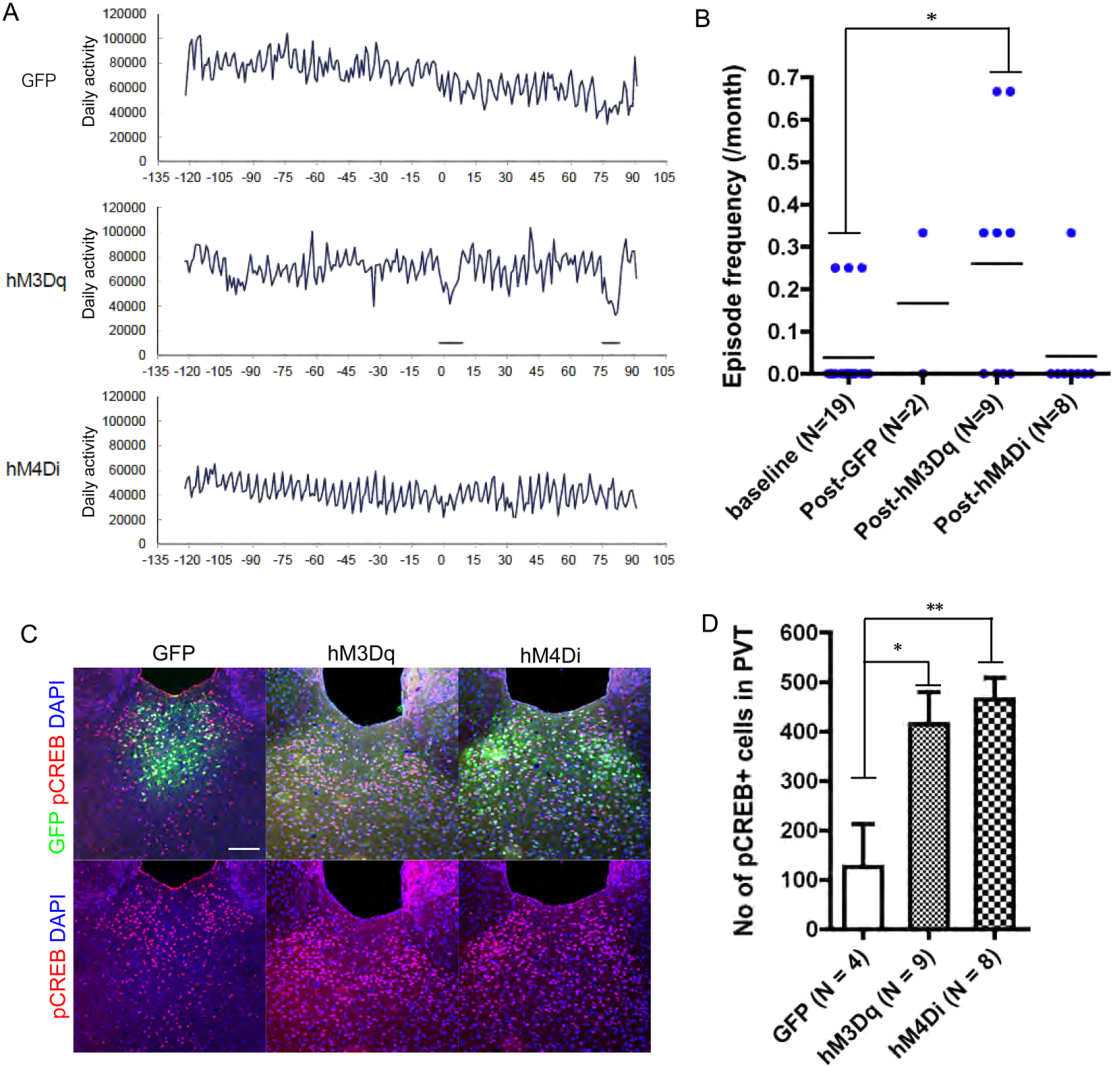
Effect of long-term manipulation of PVT neurons by DREADD on long-term wheel-running activity. (**A**) Wheel-running activity of the mice with AAV-GFP, AAV-hM3Dq, or AAV-hM4Di. Horizontal axis indicates the date from the implantation of CNO pellet (Day 0). Vertical axis shows the movement of the running wheel per day. The thick lines under the record of wheel running indicate the hypo-activity episodes. (**B**) The frequency of episodes per month before (4 months) and after (3 months) the CNO pellet implantation. *indicates *p* < 0.05 by post-hoc Dunn’s Multiple Comparison test following Kruskal-Wallis test. (**C**) images indicate anti-GFP (green) and anti-pCREB (red) immunostaining and nuclear staining with DAPI (blue) in the mice with AAV-GFP, AAV-hM3Dq, or AAV-hM4Di. Scale bar indicates 100 µm. (**D**) Number of pCREB-positive cells in the three groups, AAV-GFP, AAV-hM3Dq, or AAV-hM4Di. The AAV-GFP group included two mice housed under the LL condition, because they showed similar pCREB staining with mice under the LD condition. Error bars indicate standard error of mean. * and **indicate *p* < 0.05 and *p* < 0.01, respectively.

Next, we examined the effect of long-term modulation of the activity of PVT neurons by DREADD on long-term wheel-running activity measurements in this study. For this analysis, we generated female mice infected with AAV-hM3Dq, AAV-hM4Di or AAV-GFP and subcutaneously implanted drug-releasing pellets containing CNO in the middle of wheel-running measurements. The first 4 months of measurements were defined as baseline and the following 3 months after the implantation of CNO pellets were evaluated as a test period for the modulation of neuronal activity. The frequency of the hypoactivity episodes in the mice with AAV-hM3Dq after the CNO implantation was significantly greater than that in all mice before CNO implantation,whereas that was not significantly increased in the group of AAV-GFP and AAV-hM4Di (Fig. 2A,B). After wheel-running measurements, we conducted immunohistochemical staining for GFP and pCREB and found a significant increase in pCREB-positive cells in the PVT region in AAV-hM3Dq mice (Fig. 2C,D and Supplementary Fig. 2). These results suggest that AAV-hM3Dq mice implanted with CNO-releasing pellets showed sustained activity of PVT neurons and increased hypoactivity episodes. Unexpectedly, however, AAV-hM4Di mice also showed increased pCREB-positive cells in the PVT (Fig. 2C,D). There might be uncertain feedback mechanisms caused by chronic hM4Di activation.

### Effect of chronic presynaptic inhibition in PVT neurons by TeTX on FST and TST

**Figure 3:**
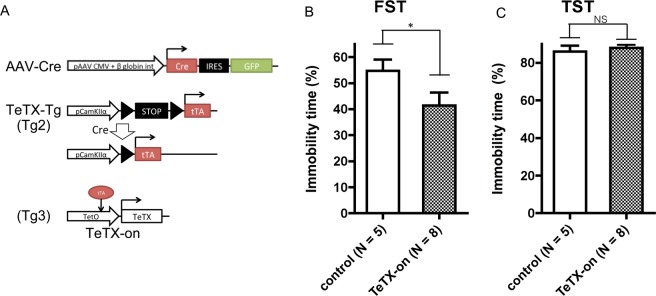
Effect of presynaptic inhibition in PVT neurons by TeTX on the FST and TST. (**A**) AAV construct and genetic background of the TeTX mice. (**B**,**C**) Bar plots showing the ratio of immobility time in total time (6 mins) in the FST (**B**) and TST (**C**). Error bars indicate SEM. * indicates *p* < 0.05 by one-tailed Student’s t-test.

We further examined the effect of chronic presynaptic inhibition in PVT neurons by TeTX on the FST and TST (Fig. 3A). The immobility time of the TeTX-expressing mice was significantly shorter than that of control mice in the FST (Fig. 3B). There was no detectable change in the TST (Fig. 3C). On the other hand, acute modulation of PVT neurons by DREADD did not affect FST and TST (Supplementary Fig. 3).

## Discussion

In rodents, several regions of the medial prefrontal cortex (mPFC) are distinguished by their distinct cytoarchitectures such as the anterior cingulate, prelimbic, or infralimbic cortex. Among them, PVT neurons are reported to project to the layers 1 and 5 of the prelimbic and infralimbic cortices in rats, and we also confirmed that the same regions were innervated by the PVT in mice (Supplementary Fig. 4). However, functional significance of the projection from the PVT to the mPFC is not understood yet.

In this study, we found that the modulation of the activity of PVT neurons affected firing patterns in mPFC neurons (Fig. 1). Chronic presynaptic inhibition in PVT neurons increased the proportions of the interneurons that showed intermittent firing patterns with reduced FR in isolated units (Fig. 1C–E and Supplementary Fig. 1A,B). In addition, activation of PVT neurons by hM3Dq expression and CNO administration affected the FR of pyramidal neurons in the mPFC, whereas the effect was nullified in the TeTX-on conditions (Fig. 1F). Although recent reports indicated that CNO or its metabolite clozapine could affect neuronal activities and animal behaviors^15,16^, the observations in the experiments in this study did not correspond with those findings, because this effect was not observed under the TeTX-on conditions, which would mean that alterations of FR in the mPFC depended on synaptic transmission of PVT neurons (Fig. 1F). In behavioral analysis, we found that long-term activation of PVT neurons by hM3Dq increased the frequency of depression-like episodes (Fig. 2) and chronic presynaptic inhibition in PVT neurons shortened the immobility time in the FST (Fig. 3).

Our electrophysiological experiments in the mPFC in the mice in which PVT neurons were manipulated by hM3Dq and TeTX revealed that the acute activation of PVT neurons caused FR change (Fig. 1F). On the other hand, chronic presynaptic inhibition in PVT neurons altered the population of active neurons that was relatively interneuron dominant compared with normal (TeTX-off) conditions and induced intermittent firing patterns (Fig. 1E and Supplementary Fig. 1A,B), even though the features obtained from the experiments under normal conditions correspond largely to previous reports described in this region^10,14,17^. Recently, another group reported that acute inhibition of the mediodorsal thalamus (MD), a region adjacent to the PVT, mostly affected interneurons in the mPFC and selective activation of parvalbumin interneurons in the mPFC by hM3Dq ameliorated not only excitatory and inhibitory imbalance in the mPFC but also cognitive deficits caused by inhibition of MD neurons^18^. The MD as well as the PVT mainly consist of glutamatergic neurons, a part of them harboring projections to similar areas in the mPFC^19,20^, and both may have similar functions to regulate the excitatory/inhibitory (E/I) balance in the mPFC, even though they seem to subserve distinct roles associated with “flexibility” or “cognition memory” in the MD, but with “affective function” or “stress response” in the PVT probably due to the different upstream in the neural circuits^20^. Meanwhile, it cannot be completely excluded that viral vector affected not only PVT but also the adjacent MD which projects to the mPFC as well.

Intermittent firing patterns with the reduction of FR in interneurons in the mPFC were also previously observed in the rats in which the substantia nigra compacta (SNc) was lesioned by 6-hydroxydopamine and that was mediated by serotonin signaling^21^. Therefore, the characteristic firing patterns caused by chronic presynaptic inhibition of PVT neurons may be the consequence of involvement of dopamine and serotonin signaling. While the PVT receives dopamine fibers from the hypothalamus (ventrorostral A10, A11, A13 and A15 cell groups) and periaqueductal gray (dorsocaudal A10), the PVT does not receive fibers from the SNc (A9) or ventral tegmental area (VTA, A10) directory. In spite of different locus, the impairments in the PVT induce intermittent firing in the mPFC as well as that in SNc/VTA may be due to direct or indirect connection to 5-HT_3_-expressing interneurons in the mPFC.

We unexpectedly found that long-term treatment of CNO in AAV-hM4Di mice increased pCREB-positive cells in PVT neurons (Fig. 2C,D), whereas acute treatment with CNO showed the tendency to decrease c-fos positive cells in the PVT (Supplementary Fig. 3A,B). The effect of hM4Di has been shown to be mediated by G-protein coupled inward rectifier K^+^ channels (GIRKs)^22^. A previous report showed that thyrotropin-releasing hormone (TRH) enhances excitability in PVT neurons through inactivation of GIRKs by decreasing their conductance^23^. Hence, chronic activation of hM4Di in PVT neurons may paradoxically activate PVT neurons in the presence of TRH. Both TeTX and hM4Di are generally used to induce loss-of-function in specific neurons; each of them, however, shows technical limitations depending on the mechanism and in the case of PVT silencing, TeTX is considered to be more beneficial.

In this study, we used hSyn promoter for expression of DREADDs and CaMKIIα promoter for expression of tetanus toxin, and thus there is a possibility that different neurons were manipulated in these experiments. Indeed, a recent study showed promoter-specific effects of viral-mediated gene transfer (hSyn promoter vs Camk2a promoter) on synaptic transmission and plasticity in hippocampus. However, the PVT in rodents consists of mainly glutamatergic neurons, and has no GABAergic neurons. The data of Allen Brain Atlas (https://portal.brain-map.org/) also shows that the PVT consists of Vglut2 positive cells with no detectable Gad1 expressing cells. Therefore, we can assume that the neurons labelled by CaMKIIα promoter and hSyn promoter do not largely differ. Indeed, our previous study, expression of tetanus toxin in PVT neurons under CaMKIIα promoter caused reduction of Vamp2 in the similar regions to the current experiment; the prelimbic and infralimbic cortices^6^. However, we cannot completely rule out that different neurons were manipulated in these experiments.

Although several reports have suggested the association between the PVT and in human patients with major depressive disorder or bipolar disorder^7^, the roles of the PVT related to manifestation or amelioration of the symptoms remain largely unknown. Many types of stress cause induction of c-fos in the PVT region^24^. Induction of c-fos by stress is reversed by antidepressants^25^, and reduction of c-fos induction in the PVT region by antidepressants is correlated with the behavioral effect of an antidepressant on the FST^26^. Although antidepressants consistently upregulate c-fos in the PVT^27^, this is an acute effect and thus may not be related to antidepressive effect. In contrast to the present finding that long-term activation of PVT neurons by hM3Dq caused hypoactivity episode, we previously reported that chronic presynaptic inhibition in PVT neurons by TeTX increased the frequency of such episodes in long-term wheel-running activity^6^. There might be the mechanism influencing PVT targets by chronic presynaptic inhibition of PVT neurons in non-cell autonomous manner. Unless innervations of PVT neurons are completely biased to inhibitory neurons or excitatory neurons, the following both scenarios could be induced by inhibition of PVT neurons: activation of excitatory neurons via suppression of inhibitory neurons and suppression of activation of excitatory neurons. Based on our results, chronic presynaptic inhibition of PVT neurons induced dominance of inhibitory neurons in the mPFC. The fact, however, does not necessarily mean that long-term activation of PVT neurons results in opposite reaction. It is also possible that alterations of synaptic plasticity like long-term potentiation or depression in mPFC neurons are involved in regulation of this circuit. Though we did not address the effects of neural transmission from PVT neurons on the activities of mPFC neurons during the mice showing depression like behavior, the fact that PVT neurons can affect the E/I balance of mPFC neurons suggest a possibility that PVT-mPFC pathway might play a role in this behavior. The results of FST and TST were not consistent between chronic inhibition by TeTX and acute inhibition by hM4Di. Because these tests were performed at 26–66 weeks old, the age might not be optimum for these analyses. Whereas chronic inhibition of PVT neurons by TeTX reduced immobility time in FST, it did not in TST. Although both tasks are used to evaluate the effect of anti-depressants in rodents, these two test have differences in many aspects such as time course and drug sensitivity and may have different neurobiological basis which may be associated with heterogeneity of psychiatric symptoms. Thus, it would be rather interesting that only FST was affected by PVT inhibition.

In this study, we found that long-term activation of the PVT increased the frequency of depression-like hypoactivity episodes in mice. This finding suggests that proper synaptic transmission of the PVT to the related targets has important roles in depression. Further detailed studies to manipulate more specific neural circuits related to the PVT would contribute to understanding the basis of mood disorders.

## Supplementary information

Supplementary Information
