# Supplementary material for: Presynaptic dysregulation of the paraventricular thalamic nucleus causes depression-like behavior
Source: Sci Rep. 2019 Nov 11;9:16506. doi: 10.1038/s41598-019-52984-y (PMC6848207; doi:10.1038/s41598-019-52984-y)
Supplement: Supplementary file 1 — Supplementary Information [file 41598_2019_52984_MOESM1_ESM.docx]

**Presynaptic dysregulation of the paraventricular thalamic nucleus causes depression-like behavior**

Tomoaki M. Kato^1,4^, Noriko Fujimori-Tonou^1^, Hiroaki Mizukami^2^, Keiya Ozawa^2^, Shigeyoshi Fujisawa^3^, Tadafumi Kato^1^

# ^1^Laboratory for Molecular Dynamics of Mental Disorders, RIKEN Center for Brain Science, Wako, Saitama, Japan

^2^Division of Genetic Therapeutics, Center for Molecular Medicine, Jichi Medical University, Shimotsuke-shi, Tochigi, Japan

**^3^**Laboratory for Systems Neurophysiology, RIKEN Center for Brain Science, Wako, Saitama, Japan

# ^4^Present address: Department of Fundamental Cell Technology, Center for iPS Cell Research and Application, Kyoto University, Kyoto, Japan

*Correspondence to:

Tadafumi Kato, M.D., Ph.D.

Laboratory for Molecular Dynamics of Mental Disorders

RIKEN Center for Brain Science

2-1 Hirosawa, Wako, Saitama 351-0198, Japan

Tel: +81-48-467-6949

FAX: +81-48-467-6947

E-mail: tadafumi.kato@riken.jp

**Supplementary Figures**

**Supplementary Figure 1**


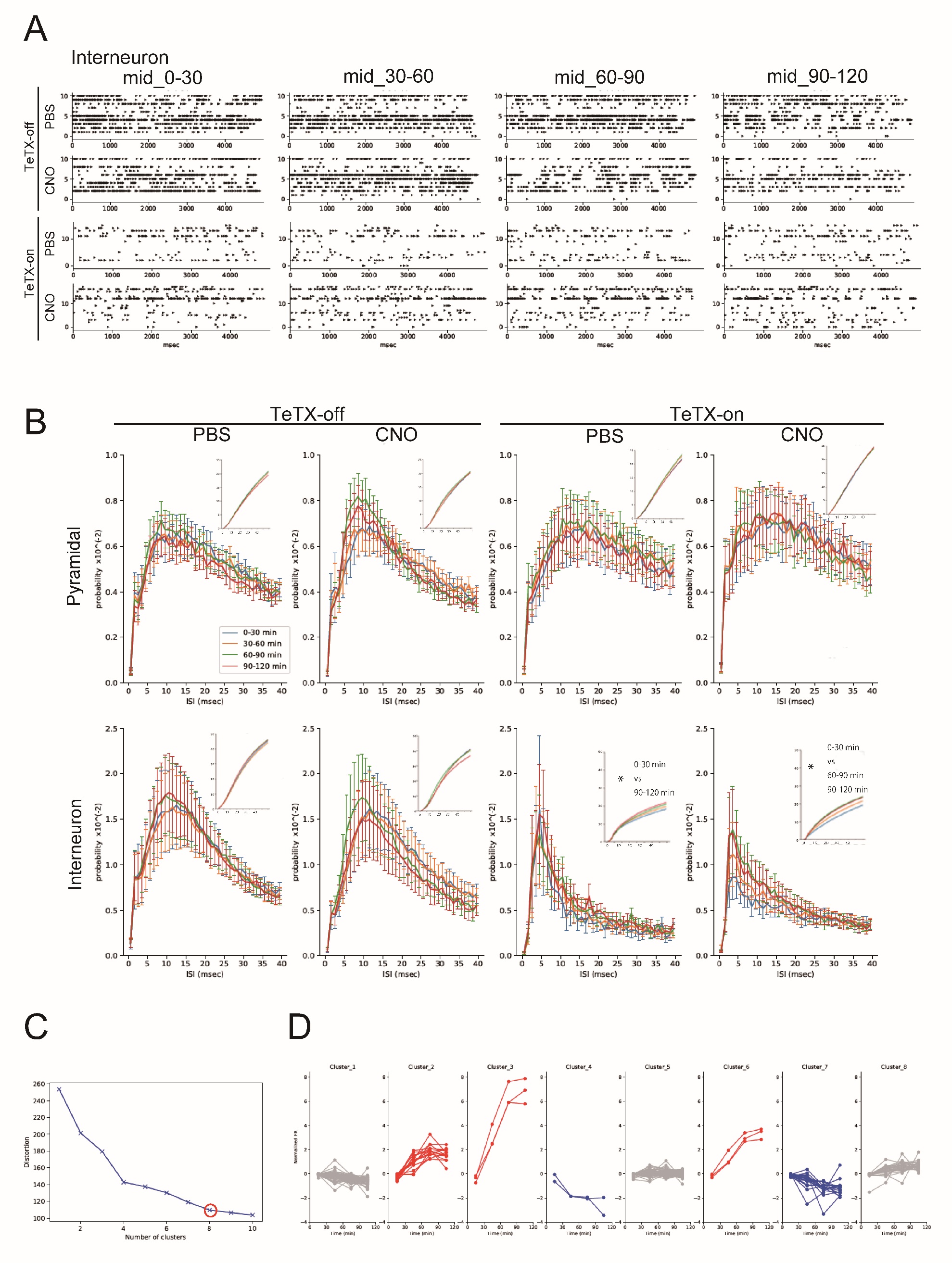


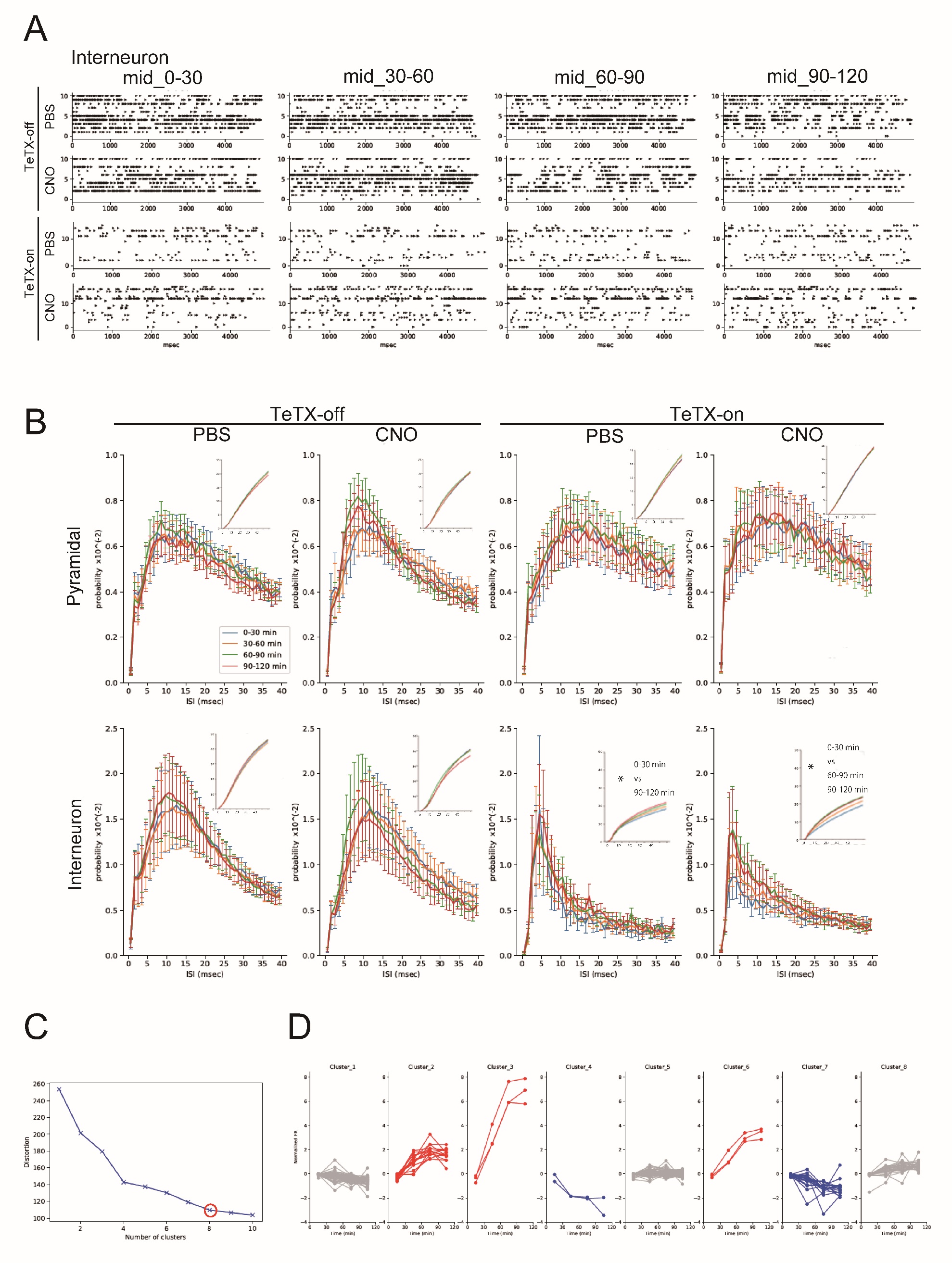


**Supplementary Figure 1. Effect of manipulation of PVT neurons on firing patterns in mPFC neurons.**

(A) Raster plots of firing activity of interneurons for 5 ms in the middle of each quarter of the sessions.

(B) Probability distributions of inter-spike intervals (ISIs) of pyramidal neurons (upper) and interneurons (bottom). The probabilities in each 1 ms-bin (main graph) and the cumulative probabilities (inset) in each quarter of the sessions are shown. Error bars indicate SEM. * indicates *p* < 0.05 by KS test.

(C) Vertical axis indicates the sum of total distance to the nearest cluster centers when the isolated units were divided into the number of clusters indicated in the horizontal axis. This graph was used for the so-called “elbow method” to estimate the optimal cluster numbers in the K means method. Over eight clusters, reduction of distortions become moderate. Therefore, the units were classified into eight clusters for further analysis.

(D) The time course of normalized FR of each unit divided into eight clusters. Red and blue indicates the units in the clusters showing increasing and decreasing FR, respectively.

**Supplementary Figure 2**


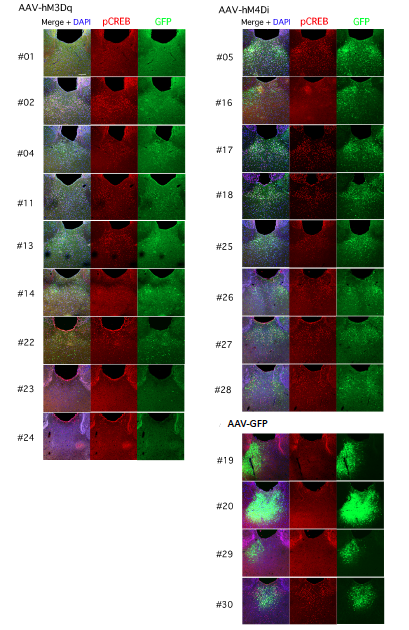


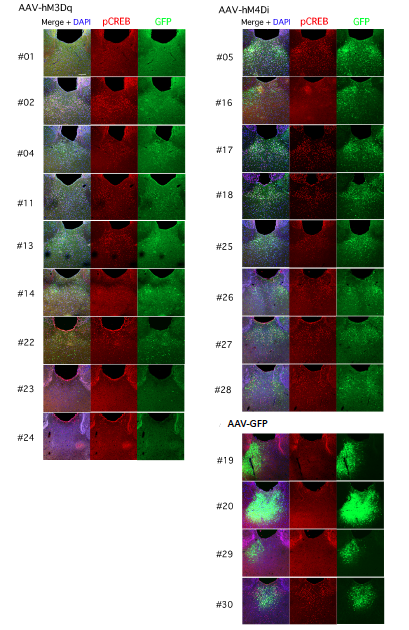


**Supplementary Figure 2. Immunohistochemical staining for GFP and pCREB after wheel-running measurement of the mice with AAV-GFP, AAV-hM3Dq and AAV-hM4Di.**

Images indicate anti-GFP and anti-pCREB immunostaining in the mice with AAV-GFP, AAV-hM3Dq, or AAV-hM4Di. Scale bar indicates 100 µm.

**Supplementary Figure 3**


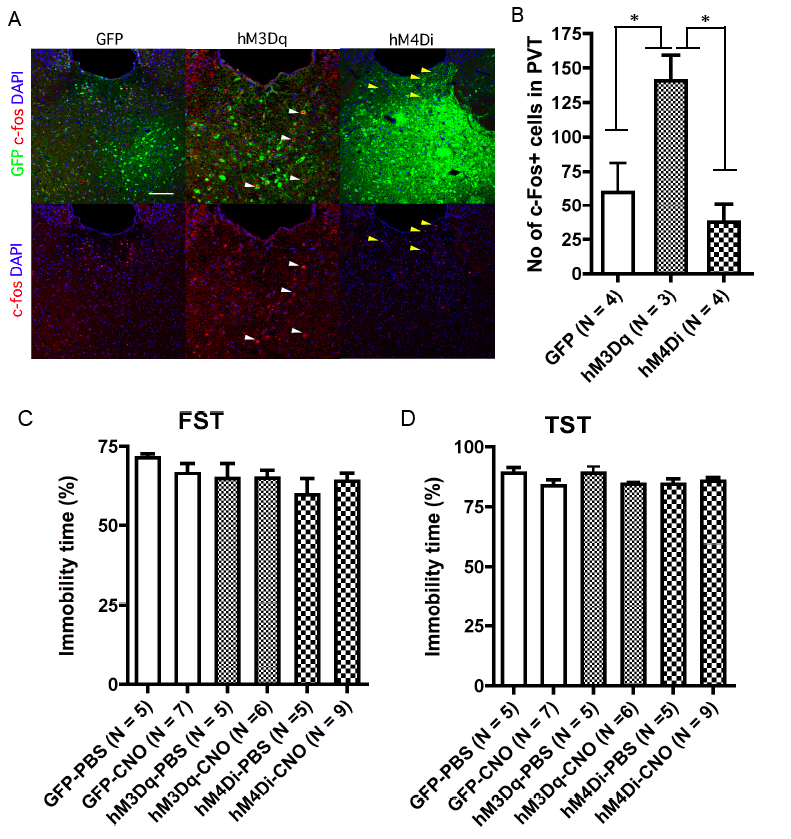


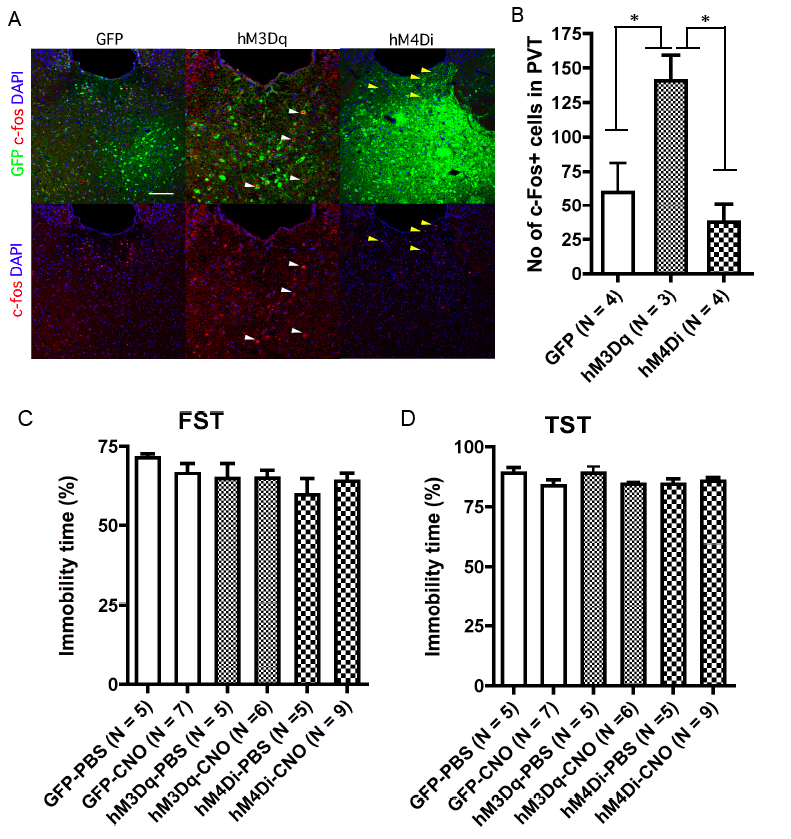


**Supplementary Figure 3.** **Effect of manipulation of PVT neurons by DREADD on the FST and TST**

(A) Immunohistochemical staining for GFP (green) and c-fos (red) in the mice infected with AAV-GFP (left panels), AAV-hM3Dq (center panels) and AAV-hM4Di (right panels). Nuclei were stained by DAPI (blue). Scale bar indicates 100 µm. White arrow heads in the center panels indicate c-fos positive cells in GFP positive cells and yellow arrow heads indicate in the right panels indicate c-fos positive cells in GFP negative cells.

(B) Bar plots showing the number of c-fos positive cells in the PVT. Error bars indicate SEM. * indicates *p* < 0.05 by post hoc Tukey’s test following one-way ANOVA.

(C and D) Bar plots showing the ratio of immobility time in total time (6 mins) in the FST (C) and TST (D). Error bars indicate SEM.

**
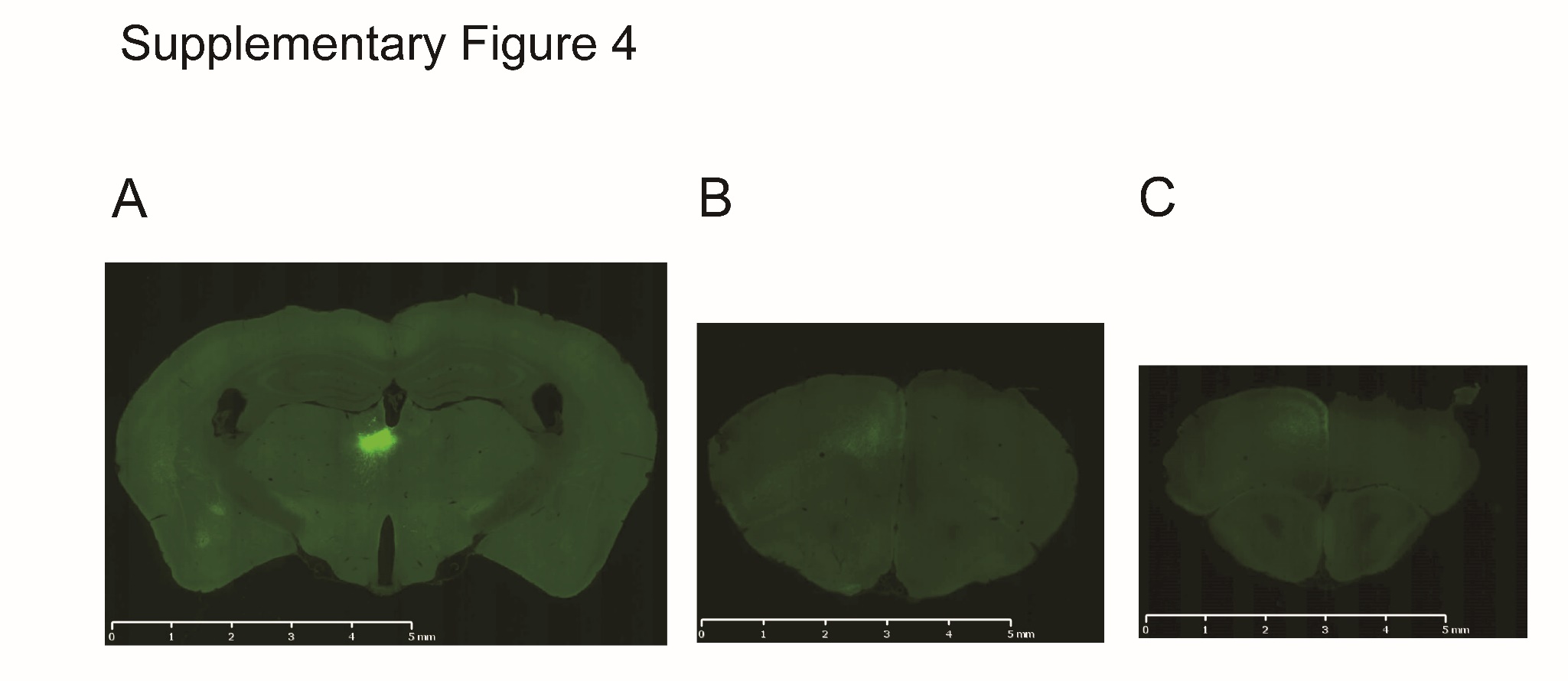
**

**Supplementary Figure 4.** **Projection of PVT neurons to medial prefrontal cortex**

(A) Injection site of AAV2-CMV-hrGFP.

(B,C) Within the medial prefrontal cortex, layers 1 and 5 of the prelimbic and infralimbic cortices are major projection site of PVT neurons.
